# Oligostyrylbenzene Derivatives with Antiparasitic and Antibacterial Activity as Potent G-Quadruplex Ligands

**DOI:** 10.3390/molecules29245875

**Published:** 2024-12-12

**Authors:** Manuel Pérez-Soto, Pablo Peñalver, Paloma Muñoz-Báez, Juan Tolosa, Joaquín Calixto García-Martínez, Rubén Cebrián, Juan Carlos Morales

**Affiliations:** 1 Departamento de Bioquímica y Farmacología Molecular, Instituto de Parasitología y Biomedicina López Neyra, CSIC, PTS Granada, Avenida del Conocimiento 17, 18016 Armilla, Spain; manuelperezsoto@ipb.csic.es (M.P.-S.); pablo@ipb.csic.es (P.P.); 2 Department of Clinical Microbiology, Instituto de Investigación Biosanitaria ibs. GRANADA, University Hospital Clínico San Cecilio, Av. de la Innovación s/n, 18061 Granada, Spain; paloma.mb11@gmail.com; 3 Department of Inorganic and Organic Chemistry and Biochemistry, Faculty of Pharmacy, Universidad de Castilla-La Mancha, C/José María Sánchez Ibáñez s/n, 02008 Albacete, Spain; juan.tolosa@uclm.es (J.T.); joaquinc.garcia@uclm.es (J.C.G.-M.); 4 CIBER de Enfermedades Infecciosas, CIBERINFEC, ISCIII, 28029 Madrid, Spain

**Keywords:** DNA, G-quadruplex, ligands, photophysical properties, antiparasitic, antibiotic

## Abstract

G-quadruplexes (G4s) are non-canonical secondary structures that play a crucial role in the regulation of genetic expression. This study explores the interaction between G4s and a small family of oligostyrylbenzene (OSB) derivatives, characterized by tris(styryl)benzene and tetrastyrylbenzene backbones, functionalized with either trimethylammonium or 1-methylpyridinium groups. Initially identified as DNA ligands, these OSB derivatives have now been recognized as potent G4 binders, surpassing in binding affinity commercially available ligands such as pyridostatin and displaying good selectivity for G4s over duplex DNA. Furthermore, OSB derivatives **1** and **2** demonstrated significant antiparasitic activity against bloodstream forms of *T. brucei* and extracellular *L. major*, with high selectivity indices when compared to MRC-5 healthy control cells. Derivatives **1** and **2** exhibited moderate biocidal effects against a range of Gram-positive and Gram-negative bacterial strains. Notably, a synergistic antibacterial effect was observed when these compounds were combined with traditional antibiotics, particularly against *Acinetobacter baumannii*, highlighting their potential utility in addressing drug-resistant bacterial infections. The differences in bioactivity among the OSB derivatives can be attributed to variations in cellular uptake, as proved by flow cytometry analysis. This suggests that the degree of cellular internalization plays a pivotal role in the observed antiparasitic and antibacterial efficacy.

## 1. Introduction

G-quadruplexes (G4s) are non-canonical nucleic acid secondary structures found in guanine-rich nucleic acid sequences. These structures have attracted significant attention due to their involvement in essential biological processes and their potential as therapeutic targets [1]. G4s are formed through the stacking of G-tetrads, which are planar arrangements of four guanine bases stabilized by Hoogsteen hydrogen bonding. The presence of monovalent cations enhances the stability of G4 structures, with the stabilization strength depending on the specific ion (K^+^ > Na^+^ > NH_4_^+^ > Li^+^) [2]. The structural diversity of G4s is notable, with various topologies observed including parallel, antiparallel or hybrid forms, which depend on the sequence composition and environmental conditions. Advances in biophysical techniques, such as nuclear magnetic resonance (NMR) spectroscopy and X-ray crystallography, have been instrumental in elucidating the structural details of these G-quadruplex formations [3].

G4s can form in both DNA and RNA and are located in various regions of the genome, including promoter regions, telomeres and untranslated regions (UTRs) of genes [2]. G4s play critical roles in regulating gene expression, maintaining genomic stability and preserving telomere integrity [4]. By inhibiting the activity of the telomerase—the enzyme responsible for telomere elongation in cancer cells—G4s present themselves as promising therapeutic targets for anti-cancer drugs. Furthermore, their presence in the promoter regions of oncogenes such as *MYC* and *KRAS* suggests that G4s may function as transcriptional regulators of genes involved in tumorigenesis [4,5]. G4 sequences have been identified not only in mammalian genomes but also across a range of other organisms, including parasites (e.g., *Trypanosoma brucei, T.cruzi, Leishmania* spp.) [6,7], yeasts (e.g., *Sacharomyces cerevisiae*) [8], bacteria (e.g., *Escherichia coli*) [9] and viruses (e.g., papilloma virus or HIV) [10].

A wide array of small molecules have emerged as promising ligands for G-quadruplexes due to their ability to selectively bind and stabilize these structures. Typically, G4 ligands feature heteroaromatic scaffolds and are surrounded by positively charged groups. These small molecules can interact with G-quadruplexes through π-π stacking with the terminal G-tetrads, electrostatic interactions with the phosphate backbone, or binding to the G4 grooves [11,12]. Several families of G4 ligands have been developed, including naphthalene diimides, porphyrins, acridines, quinozalines, pyridostatin and their derivatives [13,14]. G4 stabilization in cells leads to a specific biological response; for example, Hurley and co-workers demonstrated that stabilizing the *c-myc* G4 leads to the suppression of *c-myc* gene expression [15,16].

Oligostyrylbenzene (OSB) derivatives, characterized by their π-conjugated structure, have been studied due to their remarkable optical and photophysical properties [17,18]. In recent years, different biological applications of OSBs have also been investigated, including their use as gene transfection agents [19] or pH probes [20]. Moreover, OSBs have shown antifungal [21,22] and antimicrobial activity [23,24]. Recently, OSBs functionalized with positively charged groups at the edges of the scaffolds were found to be novel fluorescence and circular dichroism probes for ds-DNA and ds-RNA binding [25]. In the present study, we investigated the binding ability of this family of OSB derivatives to another type of secondary DNA structure: the DNA G-quadruplexes. To achieve this, we employed various biophysical techniques, including Förster resonance energy transfer (FRET) and UV-visible spectroscopy. Furthermore, we evaluated the in vitro antiparasitic and antibacterial activity of the OSB derivatives and explored their cellular uptake using flow cytometry to elucidate the mechanisms underlying their bioactivity.

## 2. Results and Discussion

### 2.1. Oligostyrylbenzene Derivatives

**Figure 1:**
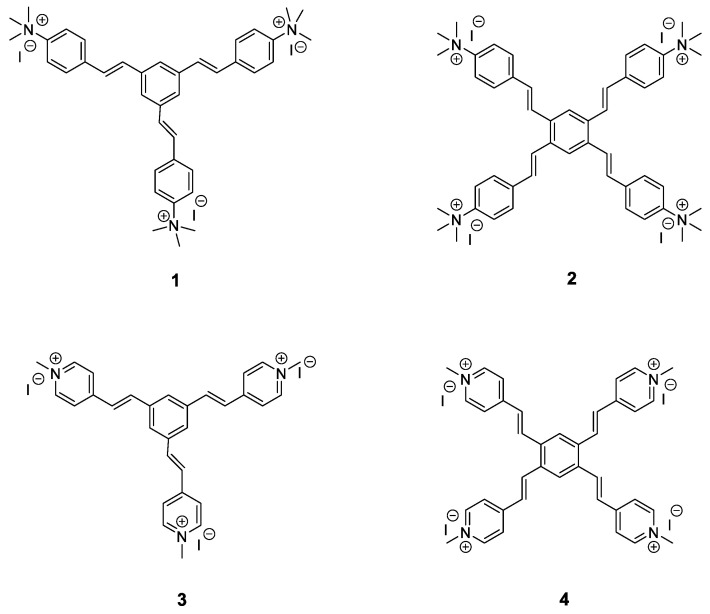
Structures of compounds **1**–**4**.

Four oligostyrylbenzene (OSB) derivatives **1**–**4** (Figure 1) were synthesized via a Horner–Wadsworth–Emmons reaction, following the methodology previously reported by Tolosa et al. [24]. This series of derivatives includes two tris(styryl)benzenes and two tetrastyrylbenzene compounds, each featuring peripheral functionalization with either trimethylammonium (**1** and **2**) or 1-methylpyridinium (**3** and **4**) units after quaternization of the dimethylamino or pyridine moiety with an excess of iodometane. In contrast to other alkenylation methods, such as the Heck coupling reaction, this approach avoids using transition metal catalysts that are difficult to remove from the samples and, even at low concentrations, may provoke interferences in biological assays.

### 2.2. Binding of OSB Derivatives to DNA Quadruplex

**Figure 2:**
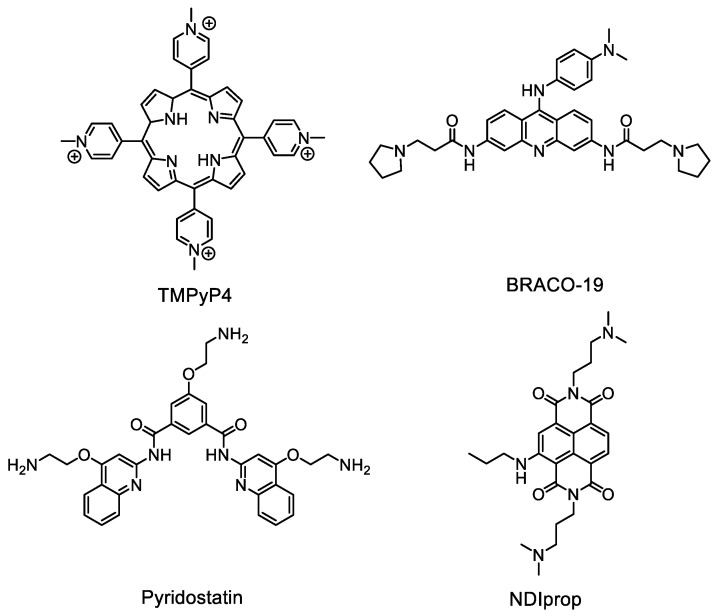
Reference compounds included in the biophysical measurements for comparison.

**Figure 3:**
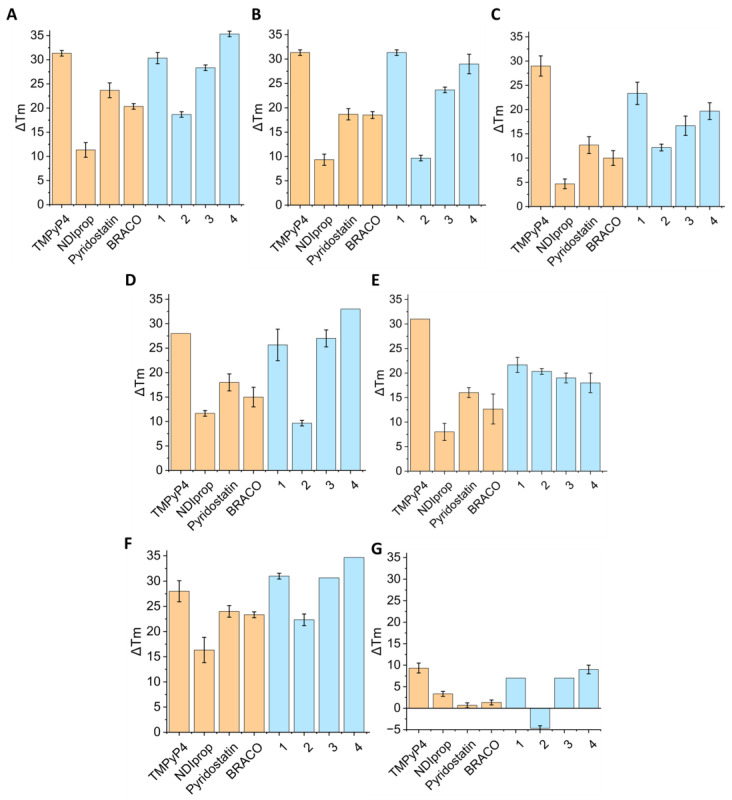
Results of the FRET-Melting Assay Performed on ligands **1**–**4** at 5 µM in blue, along with reference G4 ligands used as controls in orange (TMPyP4, NDIprop, Pyridostatin and BRACO-19) were analysed with different G4s: (**A**) F-EBR1-T, (**B**) F-SA3-T, (**C**) F-SA5-T, (**D**) F-mit6363-T, (**E**) F-mit9438-T, (**F**) F-21-T and (**G**) F-ds26-T as a duplex DNA control.

Given the general structure of these OSBs, featuring a central polyaromatic core and positively charged groups at the edges, we hypothesized that they could function as G4 ligands. To evaluate the binding affinity of the different derivatives to DNA G4s, a series of biophysical measurements were performed. First, we conducted FRET thermal melting assays with OSB derivatives **1**–**4** (at 1 and 5 μM) using fluorophore-labelled (5′-Fluorescein; 3′-TAMRA) DNA sequences, including: EBR1 G4 (hybrid G4) from *T. brucei* [6]; mit6363 and mit9438 G4s (hybrid and antiparallel G4, respectively) from human mitochondria [26]; SA-3 and SA-5 G4s (hybrid and parallel G4, respectively) from *Staphylococcus aureus* [27]; F21T G4 from the human telomere (mixed topology) [28]; and ds26 as a duplex DNA control. To benchmark the G4 binding abilities of these OSB derivatives, well-established G4 ligands such as TMPyP4, BRACO-19, Pyridostatin and NDIprop (Figure 2) were included for side-by-side comparison. In these experiments, we monitored the ligand-induced stabilisation of G4 by assessing changes in the melting temperature (ΔT_m_) of the folded sequences (Figure 3 and Figure S1).

All compounds **1**–**4** demonstrated a strong binding affinity for the different G-quadruplexes examined, with ΔT_m_ values up to 35 °C at a ligand concentration of 5 µM. Moreover, ligands **1**, **3** and **4** provided greater stabilization of the G4s compared to the reference ligands pyridostatin, BRACO-19, and NDIprop at both 1 and 5 μM concentrations. Among the new derivatives, ligand **4** exhibited the highest stabilization effect on G4s, surpassing in some cases the ΔT_m_ values measured for TMPyP4 (Figure 3 and Table S1). 

In contrast, for ds26 duplex DNA, **1**, **3** and **4** showed ΔT_m_ values of up to 9 °C at 5 µM concentration, which is comparable to the stabilizing effect observed with TMPyP4. However, compound **2** showed a notable destabilization of almost 5 °C, in contrast to previous studies where **2** showed binding to ctDNA with ΔT_m_ values exceeding 15 °C [25]. Despite this, all OSB derivatives showed a stronger affinity for G-quadruplex DNA sequences over duplex DNA, indicating a clear preference and selectivity towards G4s compared to duplex DNA.

**Figure 4:**
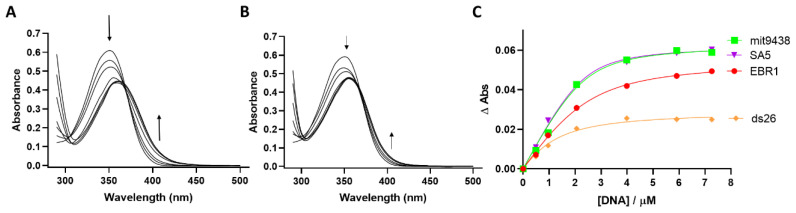
(**A**) UV-Vis spectra of ligand **3** titrated with mit9438-K^+^ and (**B**) with ds26. (**C**) UV-Vis binding isotherm illustrating the association between ligand **3** and mit9438, SA5, EBR1 and ds26 as monitored by the change in ligand absorbance at 410 nm. The arrows indicate where changes in the absorbance spectrum occur with the addition of increasing concentrations of DNA.

UV/Vis titration experiments were conducted to further validate the binding of ligands **1**–**4** to various G4 sequences and assess their selectivity for G4s over duplex DNA. The ligand concentration was fixed at 4 µM for **1** and 7 µM for **2**–**4**, while increasing concentrations of DNA were incrementally added, and the corresponding UV spectra were recorded. A pronounced bathochromic shift was observed for all G4s (Figure 4A and Figures S2–S4), in contrast to the smaller red shift observed in the absorbance maximum for duplex DNA (Figure 4B and Figure S5).

The absorbance increase (ΔAbs) at the wavelength corresponding to the new peak of the ligand–DNA complex (353 nm for **1**, 410 nm for **2**, 410 nm for **3** and 450 nm for **4**) was plotted against DNA concentration. Binding constants (K*_a_*) for OSB derivatives with three different G4 sequences (EBR1, SA5 and mit9438) and ds26 as a duplex control were then calculated using an independent-and-equivalent-sites binding model, with the best-fitting ligand:DNA stoichiometry (2:1 for ligands **1** and **2** and 3:1 for ligands **3** and **4**).

The K*_a_* values measured were in the low micromolar range for all ligands. Binding to G4s was consistently higher than that observed for duplex DNA, thus corroborating the FRET results for all the DNA sequences screened (Figure 4C and Figure S6 and Table 1).

### 2.3. Biological Activity

#### 2.3.1. Antiparasitic Activity

Given that the presence of G4s in the genome of different parasites has been previously established [6,7] and that G4s have been proposed as potential targets for antiparasitic therapies, we decided to investigate the activity of our OSB derivatives. We evaluated the effect of compounds **1**–**4** against the parasite *Trypanosoma brucei,* the causative agent of sleeping sickness [29] and *Leishmania major*, responsible for leishmaniasis [30] (Table 2). The cytotoxicity was determined in MRC-5 cell line, which comprises healthy human lung fibroblasts. Selectivity indexes (SI) were calculated (SI = IC_50_ MRC-5/IC_50_ *T. brucei* or *L. major)* to determine the therapeutic window for each compound.

Our results indicated that compounds **1** and **2**, which feature trimethylammonium groups, exhibited submicromolar and low micromolar IC_50_ values, respectively, against both *T. brucei* and *L. major* parasites. For compound **1**, the calculated therapeutic window calculated was over 100- fold and over 50-fold for *T. brucei* and *L. major* parasite, respectively. In contrast, compounds **3** and **4**, which possess 1-methylpyridinium groups, were much less active against these parasites.

When comparing OSB derivatives **1**–**4** with commercial G4 ligands, such as pyridostatin, BRACO-19 or TMPyP4, OSB **1** exhibited superior antiparasitic activity against both *T. brucei* and *L. major* as well as a broader therapeutic window. Only the naphthalene diimide-based NDIprop displayed better IC_50_ values than **1** for both parasites, though its toxicity in MRC-5 healthy cells resulted in a lower selectivity index. 

#### 2.3.2. Antibacterial Activity

Building on several studies that have highlighted the presence of G4 in bacteria [31], and with the aim of targeting these structures, we evaluated the antibacterial activity of the OSB derivatives. The minimal inhibitory concentrations (MICs) for **1**–**4** (ranging from 10 μM to 0.01 μM) were determined against four Gram-negative strains (*A. baumannii*, *E. coli*, *K. pneumoniae*, *P. aeruginosa*) and six Gram-positive strains (*B. cereus*, *E. faecalis*, *S. aureus*, *S. pyogenes*, *M. smegmatis*, *C. jeikeium*) (Table 3).

The MIC results revealed that compounds **1** and **2** (trimethylammonium derivatives) exhibit activity against two of the four Gram-negative strains tested, as well as against most of the Gram-positive strains. Notably, OSBs **1** and **2**, which had previously demonstrated antibacterial activity against *E. coli* and *E. faecalis* [24], confirmed this activity in this work. In addition, we observed even greater efficacy against other strains, such as *S. aureus*, *B. cereus* and *M. smegmatis*. In contrast, compounds **3** and **4** (1-methylpyridinium derivatives) did not show any antibacterial activity against any of the strains tested in this study. Interestingly, among the commercial G4 ligands examined—pyridostatin, BRACO-19 and TMPyP4 and the control NDIprop—BRACO-19 has been reported to display antibacterial activity, specifically against *Neisseria gonorrhoeae* [32]. TMPyP4, while photoactive against Gram-positive bacteria, was only marginally effective against Gram-negative cells [33,34]. Although NDIprop showed residual antibiotic activity against both Gram-positive and Gram-negative bacteria, an extended aromatic NDI compound did reach MIC values of up to 4–8 µM for *E. cloacae*, *B. cereus*, *E. faecalis* and *E. faecium* [27].

One of the most promising therapeutic strategies to combat bacteria infections involves the combination of drugs to obtain a synergistic effect [35]. Synergism aims to fully eradicate the infection while reducing drug dosages (and, consequently, the side effects) and shortening treatment durations. For this reason, we evaluated the combined effect of derivatives **1**–**4** with seven well-characterized antibiotics (gentamicin, meropenem, ciprofloxacin, erythromycin, rifampicin, doxycycline and vancomycin) against *S. aureus* and *A. baumannii,* the Gram-positive and Gram-negative strains where we observed the most potent antibiotic activity.

Remarkably, *A. baumannii,* a Gram-negative bacterium known for its resistance to antibiotics [36], exhibited sensitization when compounds **1**–**4** were combined with different classical antibiotics, highlighting the macrolide erythromycin—with FICI values of 0.13 to 0.38—and also the tetracycline doxycycline (FICI ranging from 0.19 to 0.25) (Table 4). Interestingly, compounds **3** and **4**, despite showing no significant antibacterial activity on their own, displayed synergism with several of the antibiotics tested, suggesting that their use in combination with conventional antibiotics could be the key to achieving an effective antibacterial response. In contrast, the synergy experiment of compounds **1**–**4** with *S. aureus* showed no evidence of synergism, despite the fact that in some cases an additive effect was observed (Table S2).

### 2.4. Cell Uptake

**Figure 5:**
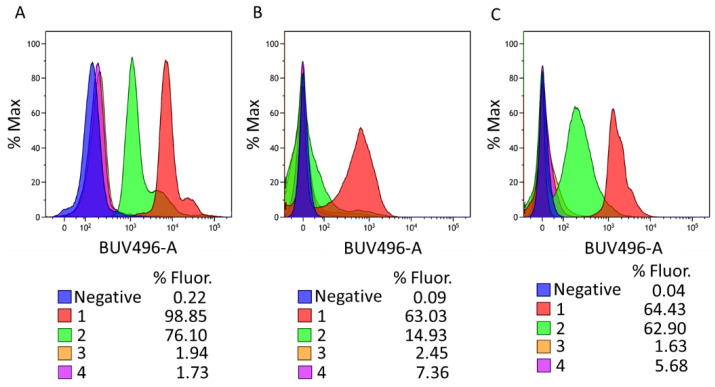
Results of cell uptake exhibited by compounds **1**–**4** at (**A**) 5 µM for 1 h at 37 °C on *T. brucei*, (**B**) 1 µM for 1 h at room temperature on *A. baumannii* and (**C**) 1 µM for 1 h at room temperature on *S. aureus*.

We observed that although compounds **3** and **4** demonstrated strong G4 binding affinity (Figure 3 and Table S1), they showed poor antiparasitic and antibiotic activity. In contrast, OSBs **1** and **2** displayed significant anti-infectious activity against both parasites and bacteria. A potential explanation for this discrepancy could be differences in cellular uptake. To investigate this, we assessed the cellular uptake of OSBs **1**–**4** using flow cytometry, taking advantage of the intrinsic fluorescence of the compounds. Flow cytometry experiments were performed on tripomastigote (*T. brucei*) parasites, which were incubated with the four compounds at 5 µM for 1 h at 37 °C. A marked difference in cellular uptake was observed among the derivatives. Compounds **1** and **2** showed high cellular uptake under the experimental conditions, with a 98.85% and 76.10% uptake, respectively. In contrast, compounds **3** and **4** exhibited minimal uptake, with values of 1.94% and 1.73%, respectively (Figure 5A).

Cell uptake experiments were also carried out on two bacterial strains: *Acinetobacter baumannii* and *Staphylococcus aureus.* Both strains were incubated with OSB derivatives **1**–**4** at 1 µM at room temperature, and flow cytometry was performed at 15 min, 30 min and 1 h of incubation (Figure 5B,C and Figure S7). Derivative **1** showed the highest cellular uptake in both bacterial strains, while derivative **2** showed a similar cellular uptake to **1** for *S. aureus*, but a slightly lower uptake in *A. baumannii*. Compounds **3** and **4**, however, showed very limited bacterial penetration, even after 1 h of incubation. These results suggest that the substantial differences in cellular uptake between the OSB derivatives likely account for the observed variations in antiparasitic and antibacterial activity. The high cellular uptake of trimethylammonium derivatives **1** and **2** explains their effective anti-infective properties, whereas the poor uptake of the 1-methylpyridinium derivatives **3** and **4** may underlie their lack of activity.

Based on these results, we propose G-quadruplexes as a potential target for this family of OSB derivatives, without discarding the possibility that these molecules may have other off-targets within the cell. Furthermore, G-quadruplexes are present not only in bacteria or parasites, but also in human cells, but different factors may cause OSB derivatives to bind more to the G4s of bacteria or parasites than to those of human cells, such as the cell cycle stage of the cell, the rate of cell division (being considerably higher for bacteria and parasites than for human cells), differences in the metabolism of the compounds or differences in cell uptake.

## 3. Experimental Section

### 3.1. General Materials and Methods

Reagents, chemicals and solvents were purchased from Sigma-Aldrich and used as supplied, without further purification. Oligonucleotides were obtained from Condalab and used without further purification. Oligonucleotide stock solutions (5 × 10 ^−4^ M) were prepared in Mili-Q water and stored at −20 °C. Oligonucleotides were annealed by incubation at 95 °C for 5 min, followed by gradual cooling to room temperature to promote G4 folding. The heating process was conducted using a thermoblock (Digital dry bath HDB120 by Labbox, Barcelona, Spain).

### 3.2. FRET-Melting Assay

FRET melting experiments were performed using the CFX Connect^TM^ real-time PCR instrument. Oligonucleotides were annealed at 10 μM in K1 buffer (1 mM KCl, 99 mM LiCl and 10 mM LiCaco, pH 7.2). The experiments were performed in 96-well plates, with each well containing 0.2 μM of oligonucleotide and compounds **1**–**4** at concentrations of 1 and 5 μM in K10 buffer (10 mM KCl, 90 mM LiCl and 10 mM LiCaco, pH 7.2), for a final volume of 25 μL.

The following dual-labelled oligonucleotides were used in the experiments: the human hairpin duplex sequence F-ds26-T (FAM-5′CAATCGGATCGAATTCGATCCGATTG3′-TAMRA), the human G4 telomeric sequence F-21-T (FAM-5′GGGTTAGGGTTAGGGTTAGGG3′-TAMRA), the parasite *T. brucei* G4 sequence F-EBR1-T (FAM-5′GGGCAGGGGGTGATGGGGAGGAGCCAGGG3′-TAMRA), *S. aureus* G4 sequences F-SA3-T (FAM-5′GGGGCTAATTGGGGCTGGTGG3′-TAMRA) and F-SA5-T (FAM-5′GGAAGGAGGGGTGACAGGG3′-TAMRA) and mitochondrial G4 sequences F-mit6363-T (FAM-5′AGGGACGCGGGCGGGGGATATAGGGT3′-TAMRA) and F-mit9438-T (FAM-5′GGCGTAGGTTTGGTCTAGGG3′-TAMRA).

FAM emissions were recorded at 516 nm, after excitation at 492 nm, in the absence and presence of the compounds as a function of temperature (25–95 °C) at a rate of 0.5 °C/min. Data were normalized between 0 and 1, where 1 represented 100% unfolding and 0 represented 100% folding, with the melting temperature (T*_m_*) corresponding to an emission value of 0.5. Each experiment was performed in duplicate or triplicate on at least two separate plates.

### 3.3. UV/Vis Spectra for Binding Studies

UV/Vis spectra were recorded using a Cary 100 UV/Vis spectrophotometer (Aligent, CA, USA) with quartz cells of 2 mm path length. Ligands were prepared at concentrations where the absorbance was higher than 0.5 (4 µM for **1** and 7 µM for **2**–**4**). First, a UV/Vis spectrum in the absence of DNA was recorded (280–500 nm). Subsequently, after annealing the different oligonucleotides at 95 °C for 5 min in K100 buffer (100 mM KCl and 10 mM LiCaco, pH 7.2), they were added at increasing concentrations, ranging from 0.07 to 1.1 times the ligand concentration, and additional UV/VIS spectra were acquired. The ligand concentration was kept constant by adding the same concentration of ligand (4 or 7 µM) to the buffer used to anneal the oligonucleotides.

### 3.4. Cell, Parasite and Bacteria Cultures

MRC-5 cells (healthy human lung fibroblasts) were cultured as a monolayer in Dulbecco’s Modified Eagle’s Medium (DMEM) with 1000 mg/L glucose and sodium bicarbonate, supplemented with 10% heat-inactivated fetal bovine serum (hiFBS, Invitrogen), 2 mM L-glutamine and 100 U/mL penicillin–100 mg/mL streptomycin. Cells were incubated at 37 °C, 100% humidity and 5% CO_2_.

*T. brucei* (bloodstream form, “single marker” S427 (S16)) were cultured in HMI-9 medium supplemented with 10% hiFBS at 37 °C and 5% CO_2_. 

*L. major* promastigotes (MHOM/IL/80/Friedlin) were cultured in modified RPMI-1640 medium (Invitrogen, Carlsbad, CA, USA) with 10% hiFBS at 28 °C and 5% CO_2_. Parasites were split every two days and maintained in the experimental growth phase at concentrations below 2 million parasites/mL.

Various Gram-negative and Gram-positive bacteria, including *Acinetobacter baumannii* ATCC 19606, *Escherichia coli* ATCC 25922, *Klebsiella pneumoniae* ATCC 700603, *Pseudomonas aeruginosa* PAO1, *Bacillus cereus* ATCC 10987, *Enterococcus faecalis* V583, *Staphylococcus aureus* ATCC 25923, Streptococcus pyogenes HUSC 263834, *Mycobacterium smegmatis* UGRA-1 and *Corynebacterium jeikeium* HUSC 223612 were grown in Mueller–Hinton broth (MHB) (*BD Difco*). The broth microdilution method was employed following the Clinical and Laboratory Standards Institute (CLSI) guidelines for aerobic bacteria [38]. When necessary, 5% of lysed horse blood was added to the medium.

### 3.5. Cytotoxicity

Cytotoxicity was determined using the Alamar blue assay (Thermo Fisher Scientific, Hillsboro, OR, USA). MRC-5 was seeded in 96-well plates (100 µL/well) at 5 × 10^4^ cells/mL in the presence of increasing concentrations of compounds **1**–**4**. After 72 h of incubation at 37 °C, 20 μL of Alamar blue solution (110 ng/mL) was added to each well, and cells were reincubated for 4 h at 37 °C. Then, 50 μL of 3% SDS Solution (sodium dodecyl sulfate in Mili-Q water) was added to each well. The plate was incubated at 37 °C for one extra hour, and fluorescence was measured using an Infinite F200 plate reader (TECAN Austria, GmbH, Grödig, Austria). The excitation wavelength was fixed at 550 nm and the emission wavelength at 590 nm. The results were expressed as the concentration of compounds that reduced cell viability by 50% (IC_50_) versus untreated control cells, calculated using SigmaPlot 16 with a four-parameter logistic curve. Data are presented as the average of three independent experiments, each conducted in triplicate.

### 3.6. Antiparasitic Activity

The trypanocidal activity of the compounds was assessed by the Alamar blue assay (Thermo Fisher Scientific). Briefly, 1 × 10^3^ of the bloodstream form of *T. brucei* were incubated in 96-well plates at 37 °C and 5% CO_2_, either alone or in the presence of increasing concentrations of compounds **1**–**4**, for 72 h. The assay was then processed as described previously. The results are expressed as the concentration of compounds that reduces parasite growth by 50% (IC_50_) compared to untreated control cells, calculated using SigmaPlot with a four-parameter logistic curve. 

The leishmaniacidal activity of the compounds on *L. major* promastigotes was determined using an MTT-based assay (Sigma-Aldrich, Merck KGaA, Darmstadt, Germany). Promastigotes (4 × 10^6^ cells/mL) were incubated in 96-well plates (50 μL/well) at 28 °C for 72 h in the presence of increasing concentrations of compounds **1**–**4**. After the incubation period, 10 μL of MTT (5 mg/mL) was added to each well, and the parasites were reincubated for 4 h at 28 °C. Then, 50 μL of 20% SDS was added to each well, and the plates were incubated at 37 °C for 4–16 h. Absorbance was measured at 540 nm using an Infinite F200 plate reader (TECAN Austria, GmbH), and the IC_50_ was calculated as described above.

All data are presented as the average of at least three independent experiments, each conducted in triplicate.

### 3.7. Antibacterial Activity

The minimal inhibitory concentration (MIC) for compounds **1**–**4**, ranging from 10 μM to 0.01 μM, was determined. Briefly, OSB derivatives were dissolved in DMSO at a stock concentration of 10 mM, and 2x serial dilutions were performed in a 96-well plate in 50 µL of MHB or MHB supplemented with 5% lysed horse blood. Subsequently, 50 µL of a bacterial suspension containing 10^5^ cells was added to each well. The plates were incubated at 37 °C for 24 h, and the MIC was defined as the lowest concentration at which no visible bacterial growth was observed.

### 3.8. Synergism Assays

For the synergism test, *Acinetobacter baumannii* ATCC 19606 and *Staphylococcus aureus* ATCC 25923 were selected as model organisms. The combined effect of gentamicin, meropenem, ciprofloxacin, erythromycin, rifampicin, doxycycline and vancomycin was evaluated. For this, 0.25× and 0.125× the MIC of compound **1**–**4** (or 2.5 µM if not active) was added to the culture medium, and the MIC of each antibiotic was determined. The MIC in the absence of the G4 ligands was used as a control. To evaluate the synergistic effect, the Fractional Inhibitory Concentration Index (FICI) was calculated following the European Committee on Antimicrobial Susceptibility Testing (EUCAST) guidelines (synergism: FICI ≤ 0.5; additive effect: 0.5 < FICI ≤ 1; indifference: 1 < FICI < 4; antagonism: FICI ≥ 4) [37]. The assay was performed in triplicate.

### 3.9. Cell Uptake

Parasites were centrifuged at 1000× *g* at 25 °C, resuspended in HMI-9 medium at a final concentration of 2 × 10^5^ BSF/mL, and incubated at 37 °C alone and in the presence of 10 μM of the different compounds for 1 h. After incubation, the parasites were centrifuged for 45 s at 12,000 rpm, the supernatant was aspirated, and the parasite pellet was resuspended in 200 μL of room-temperature phosphate-buffered saline (PBS). 

Parasites were then analysed on a FACScalibur flow cytometer (BD Biosciences, San Jose, CA, USA), acquiring 10,000 events per condition. The samples were excited at 355 nm and the emission was recorded at 496 nm. The percentage of cellular uptake was calculated using flowJo software in comparison to that of untreated cells (negative control).

For bacterial cell uptake studies, fresh MHB was inoculated at 10% with an overnight culture of *Acinetobacter baunannii* LMG01041 and *Staphylococcus aureus* LMG8224, and incubated at 37 °C with shacking until an OD600 of 0.5 was reached. The cells were washed three times in GHPES buffer (HEPES + 5 mM of glucose) and resuspended at 0.5 McFarland standards in the same buffer (1.5 × 10 ^−8^ CFU/mL). Next, the cells were incubated with 1 µM of compounds **1**–**4** for 60 min, and drug uptake was evaluated by flow cytometry as indicated before.

## 4. Conclusions

In this study, we carried out DNA binding investigations on a small family of OSB derivatives and evaluated their antiparasitic and antibacterial activity. This family includes two tris(styryl)benzenes and two tetrastyrylbenzenes, each functionalized with either trimethylammonium (compounds **1** and **2**) or 1-methylpyridinium (compounds **3** and **4**) groups. Initially identified as DNA ligands [25], these compounds have now been recognized as potent G-quadruplex ligands, exhibiting moderate selectivity for G4 structures over duplex DNA.

The biophysical experiments, including FRET and UV/vis spectroscopy, revealed significant differences in the binding of these OBS derivatives to G4 and duplex DNA. FRET experiments showed that the binding affinity to G4s was, on average, 20 °C higher than that to duplex DNA. Furthermore, the OSB derivatives demonstrated superior G4 binding compared to the well-known commercial ligands pyridostatin and BRACO-19. UV/Vis titration experiments confirmed these findings, with the binding constant (K*_a_*) being consistently higher for G4s than for duplex DNA, ranging from 2- to 10-fold differences.

Given the existence of G4 in both parasites and bacteria, we also tested the antiparasitic and antibacterial properties of these compounds. OSB derivatives **1** and **2** showed IC_50_ values in the low micromolar range against *T. brucei* and *L. major*, accompanied by high selectivity indices (SI), particularly for compound **1**, which achieved an SI of 109.8 in *T. brucei*. In contrast, compounds **3** and **4** were not toxic to parasites and their SI values were low. A similar pattern was observed for the antibacterial assays: compounds **1** and **2** were effective against several Gram-positive and Gram-negative strains, whereas compounds **3** and **4** were not. Synergistic effects were observed in experiments with *A. baumanii* and *S. aureus*. Notably, strong synergism was detected between most of the compounds and antibiotics against *A. baumannii,* even for those compounds that were inactive, which suggests an effect at any level. This synergism could potentially provide a valuable strategy for eradicating multidrug-resistant bacteria.

The observed differences in antiparasitic and antibacterial activity can be attributed to variations in cell uptake, as demonstrated by flow cytometry. Compounds **1** and **2** (active compounds) were rapidly taken up by parasites and bacteria, with uptake rates exceeding 60% in most cases after 1 h of incubation. In contrast, the cell entry for compounds **3** and **4** (non-active compounds) was very low (below 8% in all cases), which likely prevented them from reaching their intracellular target, the DNA G-quadruplexes. Altogether, our results suggest that the presence of trimethylammonium (compounds **1** and **2**) enhances cell penetration and antimicrobial activity, while the addition of an extra styrylbenzene arm (as in compound **2**), decreases the activity of this compound.

## Figures and Tables

**Table 1:** Association constants (K*_a_*) of compounds **1**–**4** with the G4 sequences EBR1, mit9438, SA5 and the ds26 duplex control. Binding constants were calculated using an independent-and-equivalent-sites binding model, with the best-fitting ligand:DNA stoichiometry.

	Ka (µM)			
	EBR1	mit9438	SA5	ds26
**1**	0.672	0.821	0.651	0.083
**2**	0.801	1.420	1.065	0.562
**3**	0.528	1.167	1.411	0.165
**4**	0.367	1.548	0.513	0.267

**Table 2:** Antiparasitic activity of OSB derivatives **1**–**4** against *T. brucei*, *L. major* and cytotoxicity values for the MRC-5 healthy human cell line. Selectivity Index (SI) values are also shown ^a^.

	IC_50_ (µM)			Selectivity Index (SI)	
	MRC-5	*T. brucei*	*L. major*	MRC-5/*T. brucei*	MRC-5/*L. major*
1	38.42 ± 0.32	**0.35 ± 0.12**	**0.70 ± 0.09**	**109.8**	**54.9**
2	80.41 ± 3.09	**3.00 ± 0.03**	10.2 ± 1.4	**26.8**	7.9
3	>100	24.06 ± 0.49	90.2 ± 6.1	>4.2	>1.1
4	>100	78.58 ± 2.48	>100	>1.3	N.D.
Pyridostatin ^b^	5.38 ± 0.07	7.82 ± 0.20	5.00 ± 0.01	0.7	1.1
BRACO-19 ^b^	8.33 ± 2.96	5.51 ± 0.99	12.73 ± 0.47	1.5	0.7
TMPyP4 ^b^	>25	>10	20.82 ± 4.86	N.D.	>1.7
NDIprop ^b^	0.36 ± 0.16	0.009 ± 0.001	0.034 ± 0.005	40.0	10.6

^a^ Data are presented as the average ± SD of three independent measurements, conducted in triplicate. Best IC_50_ and SI values are highlighted in bold. N.D. = not determined. ^b^ From reference [6].

**Table 3:** Minimal inhibitory concentration (MIC) of OSB derivatives **1**–**4** against four Gram-negative and six Gram-positive strains (shown in blue) ^a^.

	MIC (µM)			
	1	2	3	4
*Acinetobacter baumannii* ATCC 19606	2.08 ± 0.72	10	>10	>10
*Escherichia coli* ATCC 25922	5	5	>10	>10
*Klebsiella pneumoniae* ATCC 700603	>10	>10	>10	>10
*Pseudomonas aeruginosa* PAO1	>10	>10	>10	>10
*Bacillus cereus* ATCC 10987	0.83 ± 0.36	1.04 ± 0.36	>10	>10
*Enterococcus faecalis* V583	10	>10	>10	>10
*Staphylococcus aureus* ATCC 25923	0.20 ± 0.09	0.625	>10	>10
*Streptococcus pyogenes* HUSC 263834	2.5	>10	>10	>10
*Mycobacterium smegmatis* UGRA-1	1.04 ± 0.59	0.52 ± 0.18	>10	>10
*Corynebacterium jeikeium* HUSC 223612	0.83 ± 0.36	0.15	>10	>10

^a^ Data are presented as the average ± SD of three independent measurements, conducted in triplicate.

**Table 4:** Synergistic combinations for *A. baumannii*. Synergistic combinations are in bold. The concentrations of OSB derivatives **1**–**4** that exhibited the best synergistic effect are indicated. Ant., antibiotic. GN, gentamicin. MER, meropenem. CIP, ciprofloxacin. ERY, erythromycin. RIF, rifampicin. DOX, doxicicline. VAN, vancomycin ^a^.

		*A. baumannii* ATCC 19606							
		1 (0.25 µM)		2 (2.5 µM)		3 (2.5 µM)		4 (2.5 µM)	
Ant.	MIC	MIC	FICI	MIC	FICI	MIC	FICI	MIC	FICI
GN	26.7	8	**0.42**	8	**0.42**	8	**0.36**	21.333	0.86
MER	32	16	0.62	21.333	0.79	32	1.06	32	1.06
CIP	3.33	1	**0.42**	1	**0.43**	1	**0.36**	2	0.66
ERY	>64	8	**0.18**	32	**0.38**	8	**0.13**	21.333	**0.23**
RIF	2	0.5	**0.37**	0.25	**0.25**	0.5	**0.31**	0.333	**0.23**
DOX	0.125	0.016	**0.25**	0.016	**0.25**	0.016	**0.19**	0.016	**0.19**
VAN	64	64	1.12	64	1.13	32	0.56	32	0.56

^a^ The Fractional Inhibitory Concentration Index (FICI) was calculated following the European Committee on Antimicrobial Susceptibility Testing (EUCAST) guidelines [37].

## Data Availability

The data presented in this study are available upon request from the corresponding author.

## Supplementary Materials

The following supporting information can be downloaded at: https://www.mdpi.com/article/10.3390/molecules29245875/s1, Figure S1: Results of the FRET-Melting Assay at 1 μM ligand concentration; Figure S2: UV-Vis spectra of ligand (A) 1, (B) 2, (C) 3 and (D) 4 titrated with mit9438-K+ at the concentrations shown in the figure; Figure S3: UV-Vis spectra of ligand (A) 1, (B) 2, (C) 3 and (D) 4 titrated with SA5-K+ at the concentrations shown in the figure; Figure S4: UV-Vis spectra of ligand (A) 1, (B) 2, (C) 3 and (D) 4 titrated with EBR1-K+ at the concentrations shown in the figure; Figure S5: UV-Vis spectra of ligand (A) 1, (B) 2, (C) 3 and (D) 4 titrated with ds26-K+ at the concentrations shown in the figure; Figure S6: UV-Vis binding isotherm for the association between ligand (A) 1, (B) 2, (C) 4 and mit9438, SA5, EBR1 and ds26 following the change in ligand absorbance at (A) 350 nm, (B) 410 nm and (C) 450 nm; Figure S7: Results of cell uptake exhibited by compounds **1**–**4** at 1 µM for 15, 30 and 60 min at room temperature on (A) *A. baumannii* and (B) *S.aureus*; Table S1: Results of the FRET-Melting Assay carried out with ligands **1**–**4** at 5 µM; Tabla S2: Synergistic combinations for *S.aureus*.
