# Supplementary material for: Oligostyrylbenzene Derivatives with Antiparasitic and Antibacterial Activity as Potent G-Quadruplex Ligands
Source: Molecules. 2024 Dec 12;29(24):5875. doi: 10.3390/molecules29245875 (PMC11679705; doi:10.3390/molecules29245875)
Supplement: Supplementary file 1 [file molecules-29-05875-s001.zip › molecules-3294836-supplementary.pdf]

SUPPORTING INFORMATION

Oligostyrylbenzene derivatives as potent G-quadruplex ligands with antiparasitic and antibacterial activity.

Manuel Pérez-Soto, Pablo Peñalver, Paloma Muñoz Báez, Juan Tolosa, Joaquín Calixto García-Martínez, Rubén Cebrián,* Juan Carlos Morales*

Index

Figure S1. Results of the FRET-Melting Assay at 1μM ligand concentration.

Figure S2. UV-Vis spectra of ligand (A) 1, (B) 2, (C) 3 and (D) 4 titrated with mit9438-K+ at the concentrations shown in the figure.

Figure S3. UV-Vis spectra of ligand (A) 1, (B) 2, (C) 3 and (D) 4 titrated with SA5-K+ at the concentrations shown in the figure.

Figure S4. UV-Vis spectra of ligand (A) 1, (B) 2, (C) 3 and (D) 4 titrated with EBR1-K+ at the concentrations shown in the figure.

Figure S5. UV-Vis spectra of ligand (A) 1, (B) 2, (C) 3 and (D) 4 titrated with ds26-K+ at the concentrations shown in the figure.

Figure S6. UV-Vis binding isotherm for the association between ligand (A) 1, (B) 2, (C) 4 and mit9438, SA5, EBR1 and ds26 following the change in ligand absorbance at (A) 350 nm, (B) 410 nm and (C) 450 nm.

Figure S7. Results of cell uptake exhibited by compounds **1**-**4** at 1 µM for 15, 30 and 60 min at room temperature on (A) *A. baumannii* and (B) *S.aureus*.

Table S1. Results of the FRET-Melting Assay carried out with ligands **1**-**4** at 5 µM.

Tabla S2. Synergistic combinations for *S.aureus*.

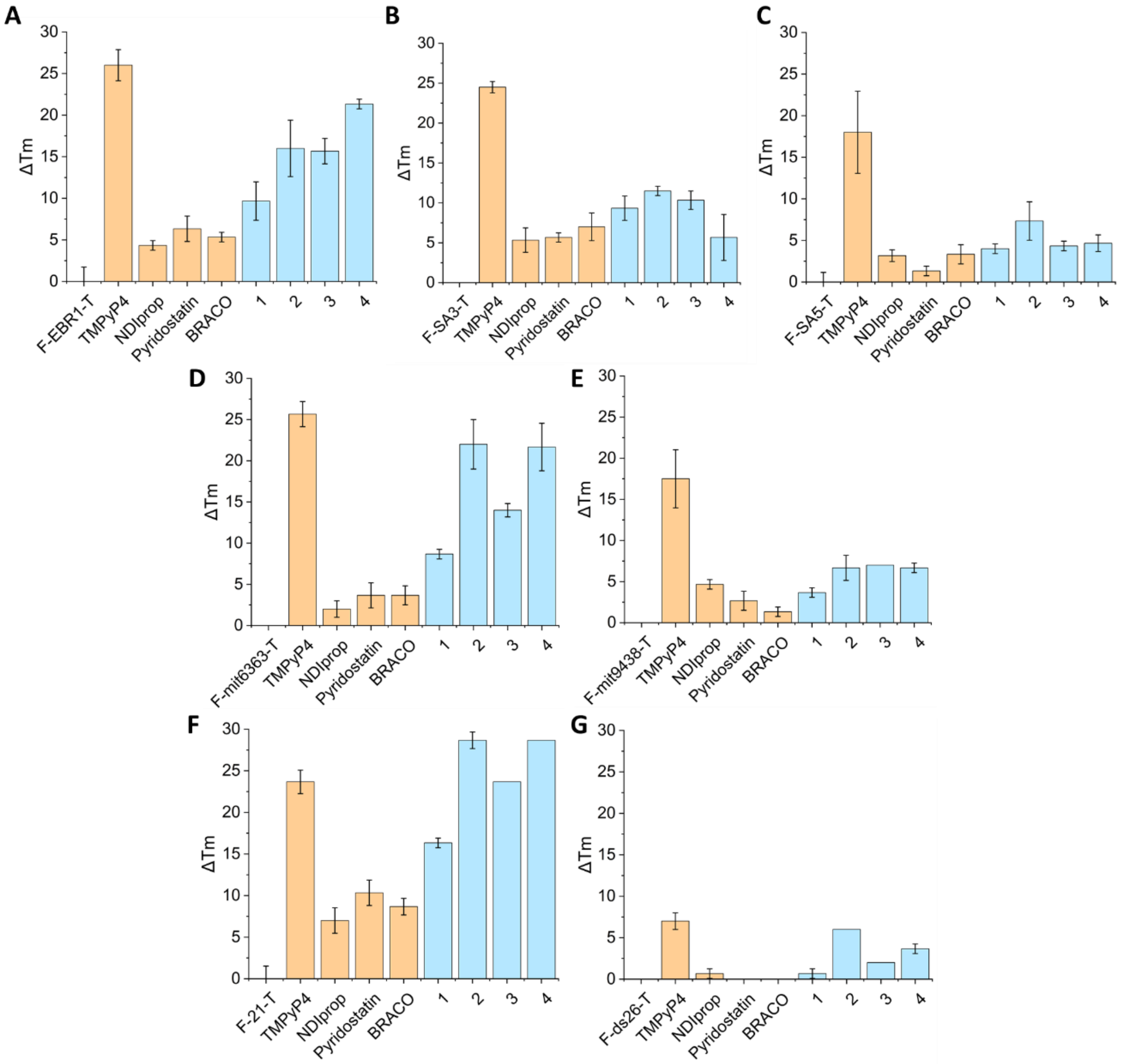

Figure S1. Results of the FRET-Melting Assay Performed on ligands **1**-**4** at 1 μM and the commercial G4 ligands (TMPyP4, NDIprop, Pyridostatin and BRACO-19) as reference with different G4s: (A) F-EBR1-T, (B) F-SA3-T, (C) F-SA5-T, (D) F-mit6363-T, (E) F-mit9438-T, (F) F-21-T and (G) F-ds26-T as a DNA control duplex.

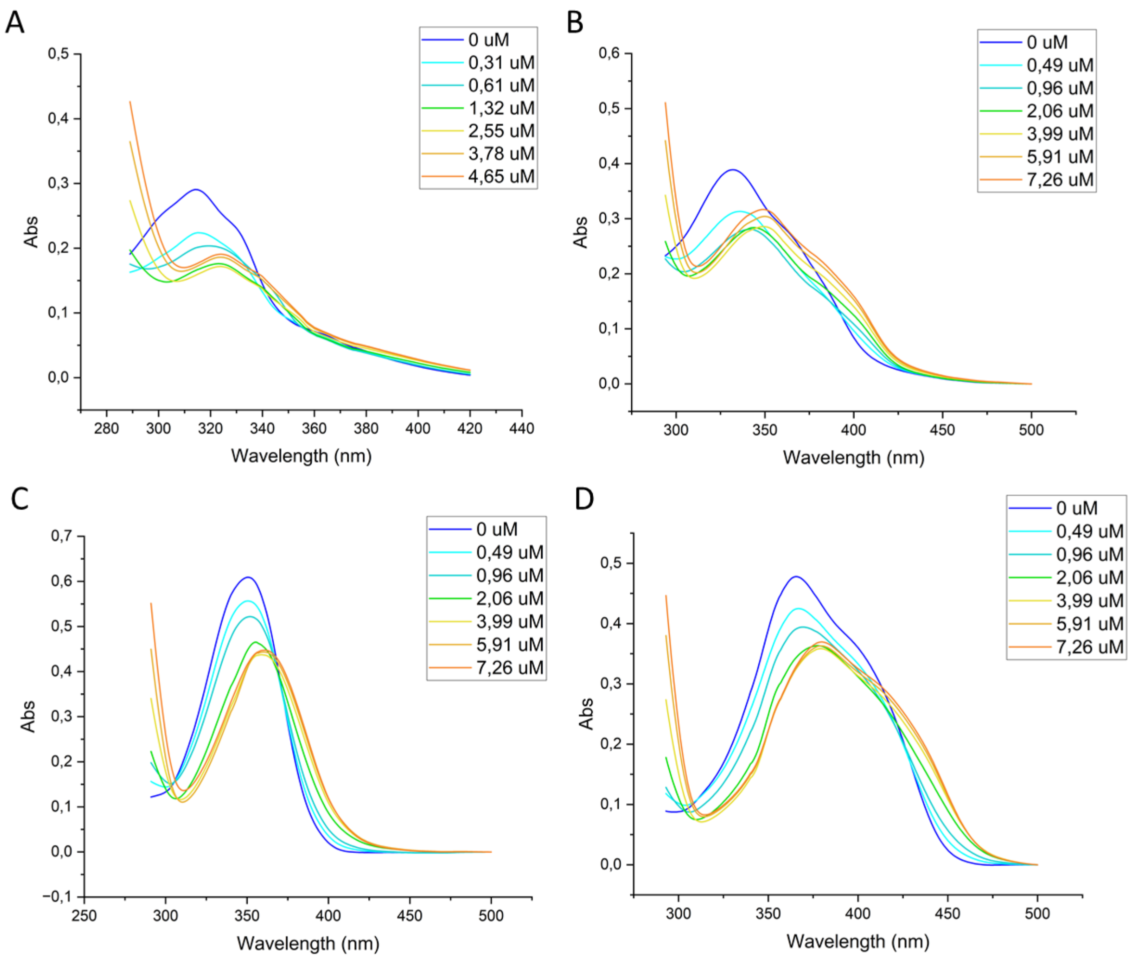

Figure S2. UV-Vis spectra of ligand (A) **1**, (B) **2**, (C) **3** and (D) **4** titrated with mit9438-K+ at the concentrations shown in the figure.

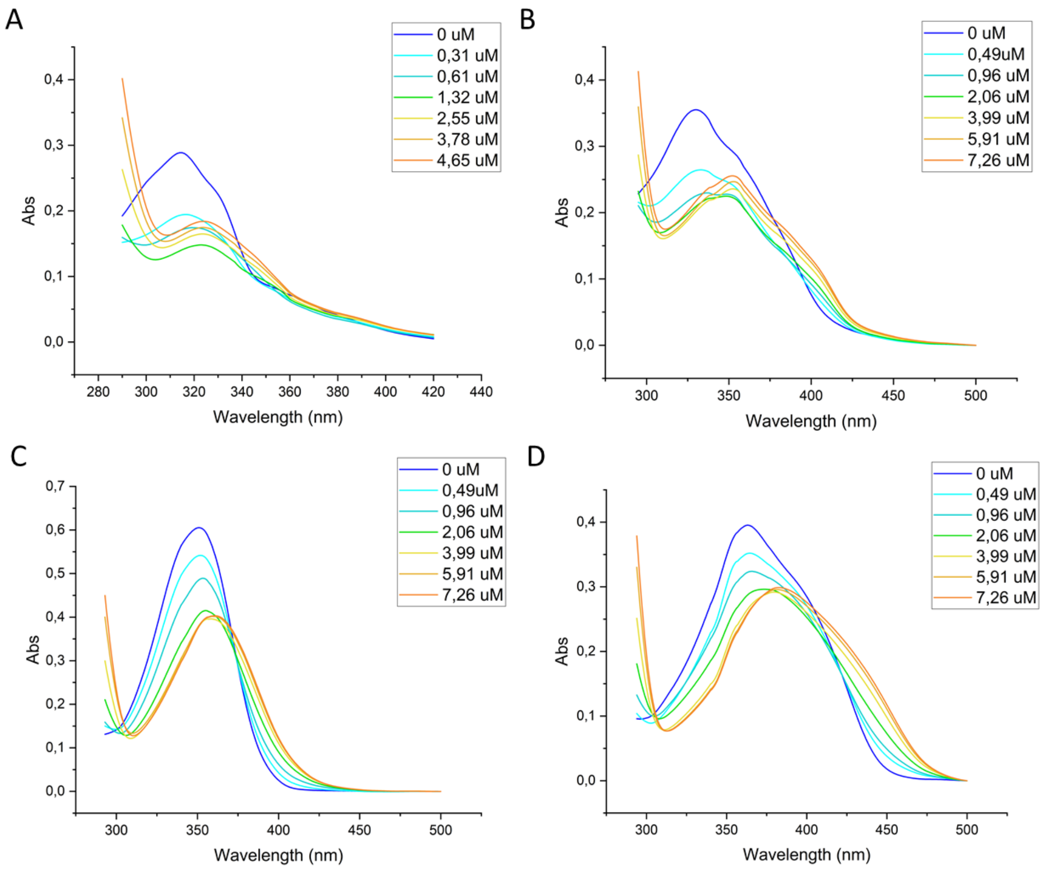

Figure S3. UV-Vis spectra of ligand (A) **1**, (B) **2**, (C) **3** and (D) **4** titrated with SA5-K+ at the concentrations shown in the figure.

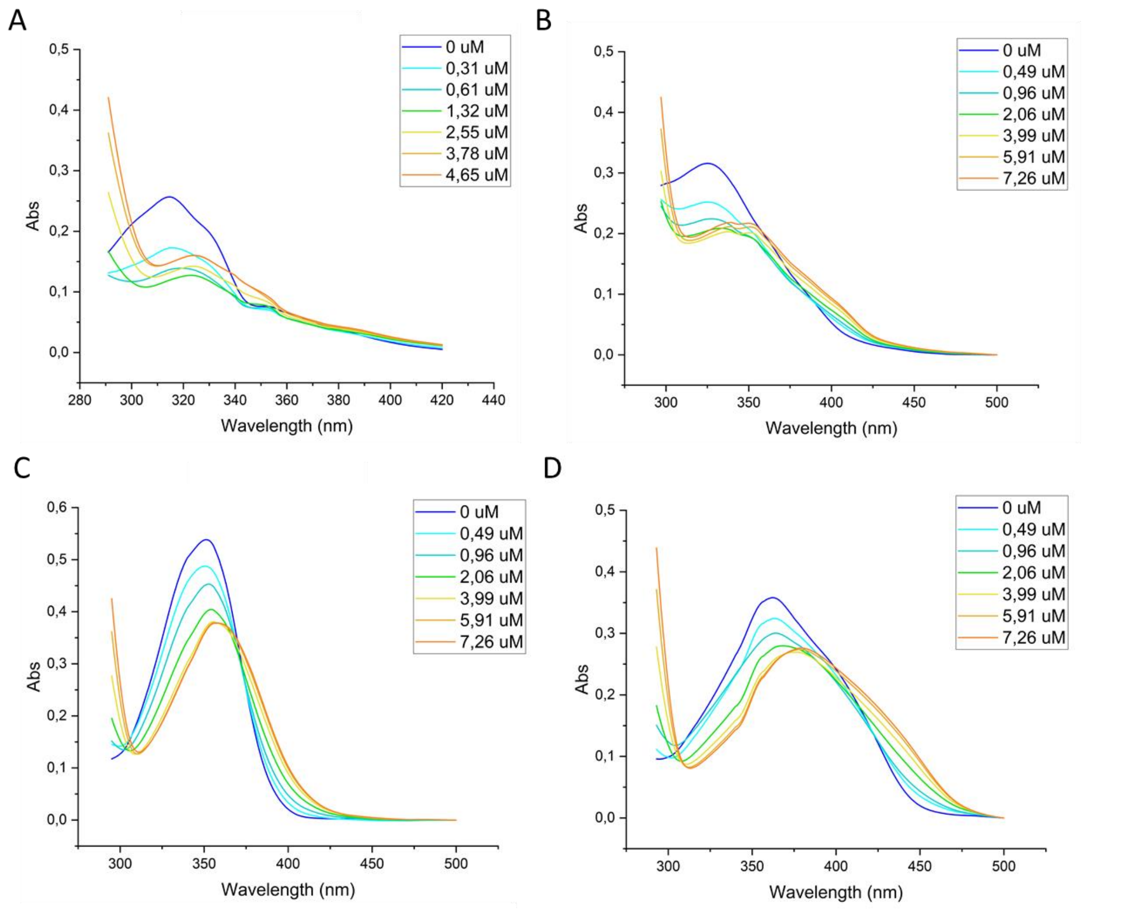

Figure S4. UV-Vis spectra of ligand (A) **1**, (B) **2**, (C) **3** and (D) **4** titrated with EBR1-K+ at the concentrations shown in the figure.

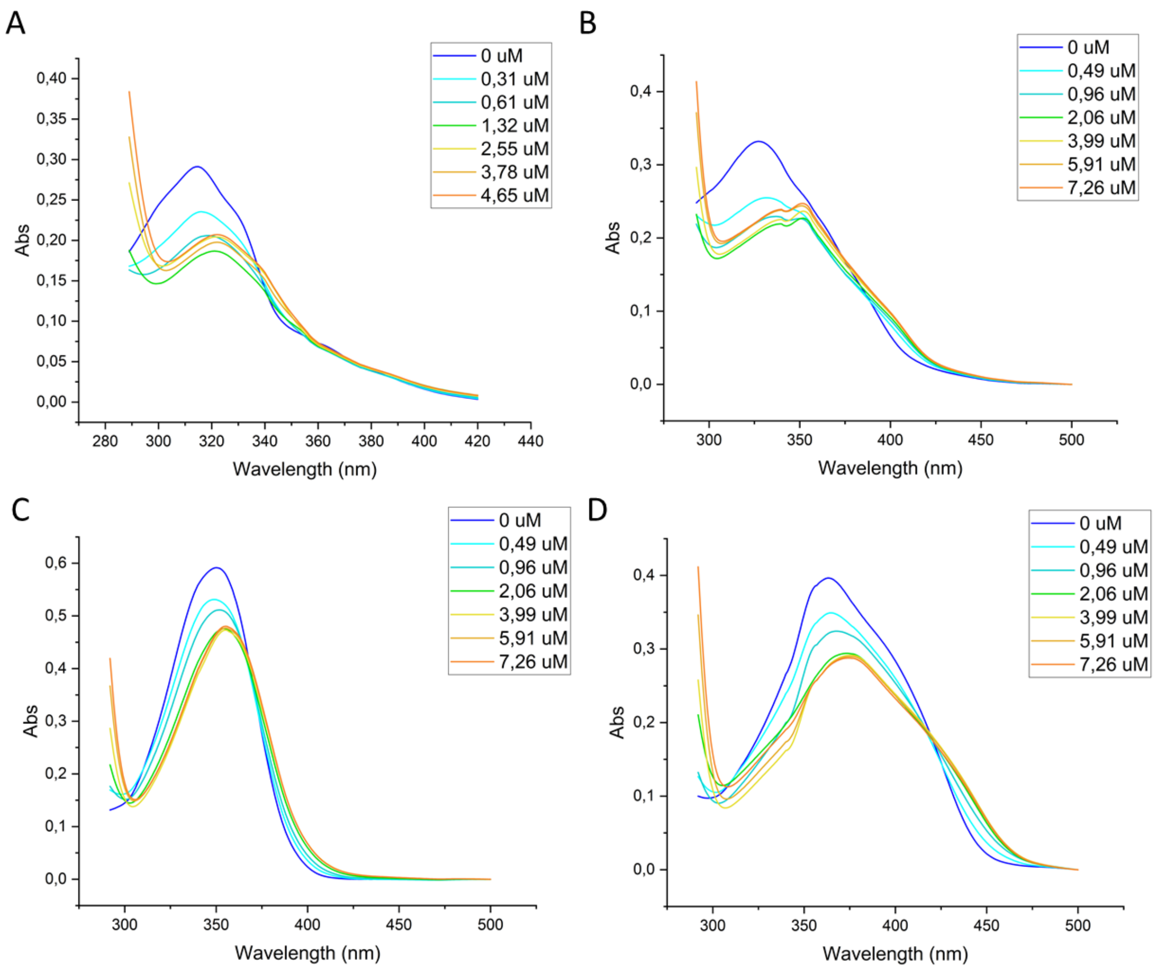

Figure S5. UV-Vis spectra of ligand (A) **1**, (B) **2**, (C) **3** and (D) **4** titrated with ds26-K+ at the concentrations shown in the figure.

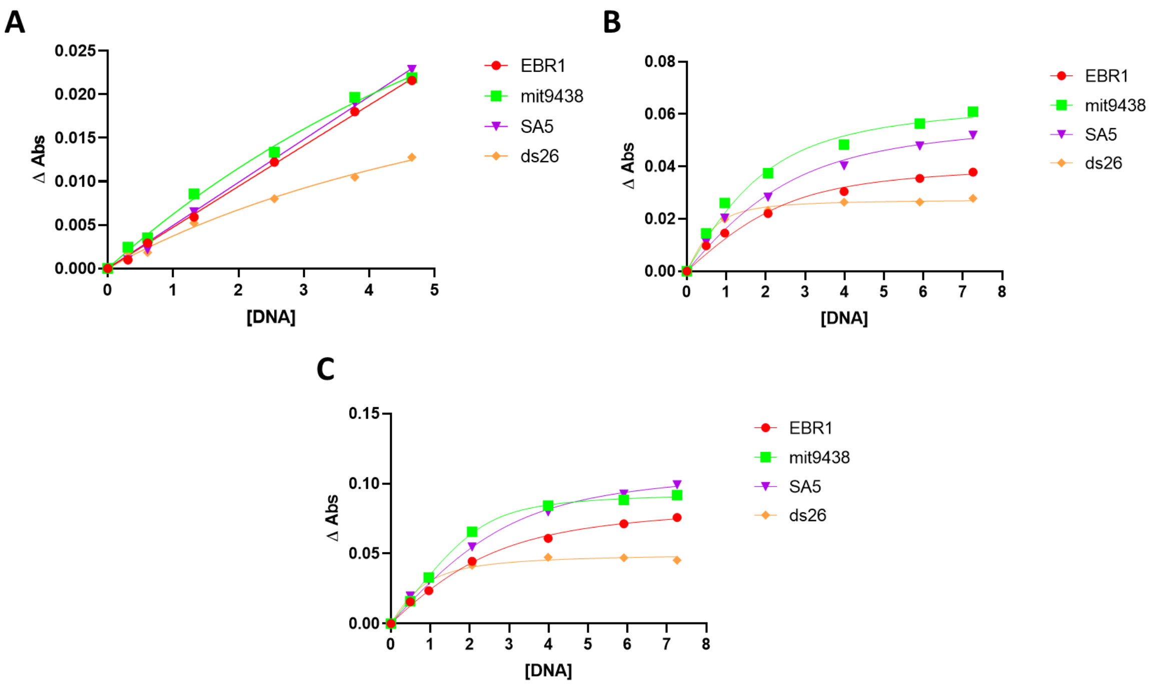

Figure S6. UV-Vis binding isotherm for the association between ligand (A) **1**, (B) **2**, (C) **4** and mit9438, SA5, EBR1 and ds26 following the change in ligand absorbance at (A) 350 nm, (B) 410 nm and (C) 450 nm.

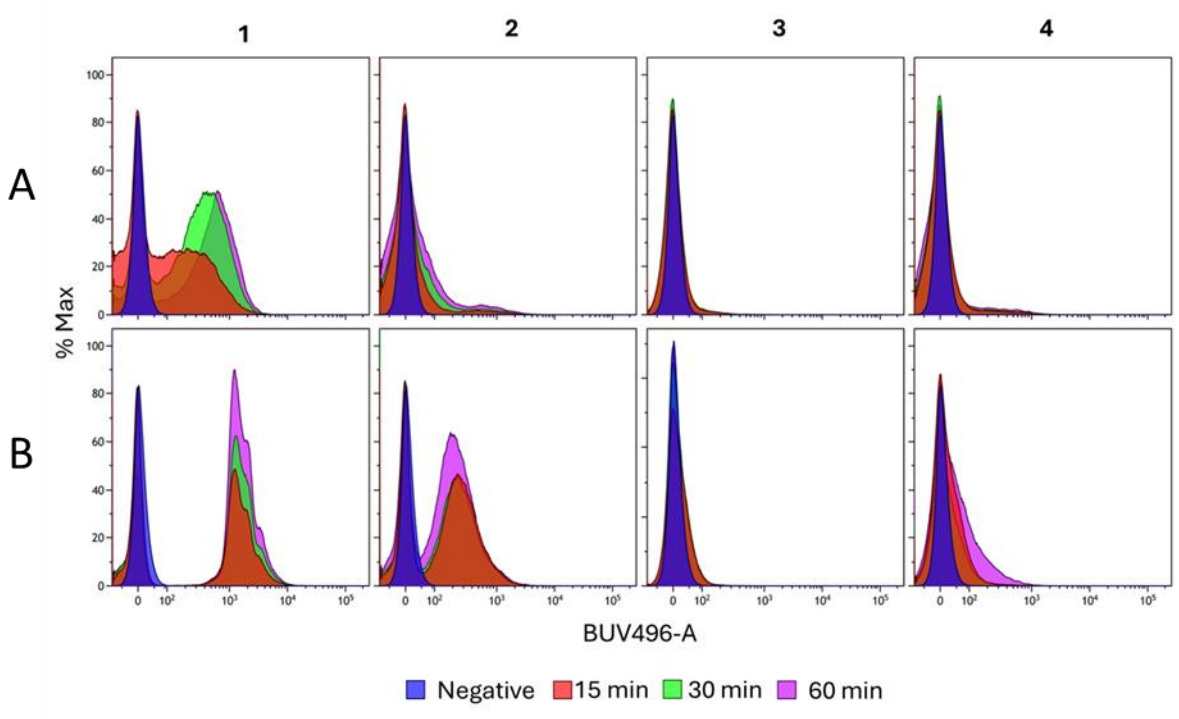

Figure S7. Results of cell uptake exhibited by compounds **1**-**4** at 1 μM for 15, 30 and 60 min at room temperature on (A) *A. baumannii* and (B) *S.aureus*.

Table S1. Results of the FRET-Melting Assay carried out with ligands **1**-**4** at 5 µM, alongside reference G4 ligands (TMPyP4, NDIprop, Pyridostatin and BRACO-19) used as controls. The assays were performed with different G4s: F-EBR1-T [FAM-5´GGGCAGGGGGTGATGGGGAGGAGCCAGGG3´-TAMRA], F-SA3-T [FAM-5´GGGGCTAATTGGGGCTGGTGG3´-TAMRA], F-SA5-T [FAM-5´GGAAGGAGGGGTGACAGGG3´-TAMRA], F-mit6363-T [FAM-5´AGGGACGCGGGCGGGGGATATAGGGT3´-TAMRA], F-mit9438-T [FAM-5´GGCGTAGGTTTGGTCTAGGG3´-TAMRA], F-21-T [FAM-5´GGGTTAGGGTTAGGGTTAGGG3´-TAMRA] and F-ds26-T [FAM-5´CAATCGGATCGAATTCGATCCGATTG3´-TAMRA], which served as a duplex DNA control. The results are expressed as ΔT_{m}.

	**EBR1**	**SA3**	**SA5**	**mit6363**	**mit9438**	**F21T**	**ds26**
TMPyP4	31.3 ± 0.6	31.3 ± 0.6	29.0 ± 2.1	28.0 ± 0.0	31.0 ± 0.0	28.0 ± 2.1	9.3 ± 1.1
NDIprop	11.3 ± 1.5	9.3 ± 1.1	4.7 ± 1.0	11.7 ± 0.6	8.0 ± 1.7	16.3 ± 2.5	3.3 ± 0.6
Pyridostatin	23.6 ± 1,5	18.7 ± 1.1	12.7 ± 1.7	18.0 ± 1.7	16.0 ± 1.0	24.0 ± 1.1	0.7 ± 0.6
BRACO-19	20.3 ± 0.6	18.5 ± 0.7	10.0 ± 1.5	15.0 ± 2.0	12.7 ± 3.0	23.3 ± 0.6	1.3 ± 0.6
**1**	30.3 ± 1.1	31.3 ± 0.6	23.3 ± 2.3	25.7 ± 3.2	21.7 ± 1.5	31.0 ± 0.6	7.0 ± 0.0
**2**	18.7 ± 0.6	9.7 ± 0.6	12.2 ± 0.7	9.7 ± 0.6	20.3 ± 0.6	22.3 ± 1.1	-4.7 ± 0.6
**3**	28.3 ± 0.6	23.6 ± 0.6	16.7 ± 2.0	27.0 ± 1.7	19.0 ± 1.0	30.7 ± 0.0	7.0 ± 0.0
**4**	35.3 ± 0.6	29.0 ± 2.0	19.7 ± 1.7	33.0 ± 0.0	18.0 ± 2.0	34.7 ± 0.0	9.0 ± 1.0

Table S2. Synergistic combinations for *S.aureus*. Shaded in light grey, synergistic combinations. The tested concentration of **1** - **4** with the best synergistic effect is indicated. Ant., antibiotic. GN, gentamicin. MER, meropenem. CIP, ciprofloxacin. ERY, erythromycin. RIF, rifampicin. DOX, doxicicline. VAN, vancomycin. [a]

		***S. aureus* ATCC25923**							
		**FL65 (0.05 µM)**		**FL67 (0.156 µM)**		**FL68 (2.5 µM)**		**FL69 (2.5 µM)**	
**Ant.**	**MIC**	**MIC**	**FICI**	**MIC**	**FICI**	**MIC**	**FICI**	**MIC**	**FICI**
GN	0.25	0.130	0.76	0.156	0.87	0.130	0.65	0.130	0.65
MER	8	8.333	1.28	8.333	1.29	6.667	0.96	6.667	0.96
CIP	1	0.313	0.55	0.833	1.08	0.833	0.96	0.313	0.44
ERY	0.375	0.313	1.07	0.417	1.36	0.417	1.24	0.417	1.24
RIF	0.004	0.002	0.73	0.003	1.06	0.004	1.10	0.002	0.61
DOX	0.25	0.156	0.86	0.313	1.50	0.208	0.96	0.156	0.75
VAN	0.67	0.521	1.02	0.521	1.03	0.625	1.06	0.417	0.75

[a] The Fractional Inhibitory Concentration Index (FICI) was calculated following the European Committee on Antimicrobial Susceptibility Testing (EUCAST) guidelines (Cebrián et al., 2021).
